# Preference and uptake of different community-based HIV testing service delivery models among female sex workers along Malaba-Kampala highway, Uganda, 2017

**DOI:** 10.1186/s12913-019-4610-3

**Published:** 2019-11-05

**Authors:** Gerald Pande, Lilian Bulage, Steven Kabwama, Fred Nsubuga, Peter Kyambadde, Shaban Mugerwa, Joshua Musinguzi, Alex Riolexus Ario

**Affiliations:** 1 Uganda Public Health Fellowship Program, Kampala, Uganda; 2 AIDS Control Program, Kampala, Uganda; 3 African Field Epidemiology Network, Kampala, Uganda; 4 Ministry of Health, Kampala, Uganda

**Keywords:** Key populations, HIV testing, Linkage to care, Sex workers, Uganda

## Abstract

**Introduction:**

Female Sex workers (FSW) and their clients accounted for 18% of the new HIV infections in 2015/2016. Special community-based HIV testing service delivery models (static facilities, outreaches, and peer to peer mechanism) were designed in 2012 under the Most At Risk Populations Frame work and implemented to increase access and utilization of HIV care services for key populations like female sex workers. However, to date there is no study that has been done to access the preference and uptake of different community-based HIV testing service delivery models used to reach FSW. We assessed preference and uptake of the current community-based HIV testing services delivery models that are used to reach FSW and identified challenges faced during the implementation of the models.

**Methods:**

We conducted a cross-sectional study design using quantitative (interview with the health workers in facilities providing services to female sex workers and interviews with FSWs) and qualitative (interviews with Ministry of Health staff, health workers, district health team members, program staff at different levels involved in delivery of HIV care services, FSWs and political leaders to assess for the enabling environment created to deliver the different community-based HIV testing services to FSWs along the Malaba-Kampala highway. Malaba – Kampala high way is one of the major high ways with many different hot spots where the actual buying and selling of sex takes place. We defined FSWs as any female, who undertakes sexual activity after consenting with a man for money or other items/benefits as an occupation or as a primary source of livelihood irrespective of site of operation within the past six months.

We assessed the preference and uptake of different community based HIV testing services delivery model among FSWs based on two indicators, i.e., the proportion of FSWs who had an HIV Counseling and Testing (HCT) in the last 12 months and the proportion of FSWs who were positive and linked to care.

**Results:**

Overall, 86% (390/456) of the FSWs had taken an HIV test in the last 12 months. Of the 390 FSWs, 72% (279/390) had used static facilities, 25% (98/390) had used outreaches, and 3.3% (13/390) used peer to peer mechanisms to have an HIV test. Overall, 35% (159/390) of the FSWs who had taken an HIV test were HIV positive. Of the 159, 83% (132/159) were successfully linked into care. Ninety one percent (120/132) reported to have been linked into care by static facilities. Challenges experienced included; lack of trust in the results given during outreaches, failure to offer other testing services including hepatitis B and syphilis during outreaches, inconsistent supply of testing kits, condoms, STI drugs, and unfriendly health services due to the infrastructure and non-trained health workers delivering KP HIV testing services.

**Conclusions:**

Most of the FSWs had HCT services and were linked to care through static facilities. Community-based HIV testing service delivery models are challenged with inconsistent supply of HIV testing commodities and unfriendly services.. We recommended strengthening of all HIV testing community-based HIV testing service deliverymodels by ensuring constant supply of HIV testing/AIDS care commoditiesoffering FSW friendly services, and provision of comprehensive HIV/AIDS health care package.

## Introduction

According to the Joint United Nations Programme on HIV and AIDS (UNAIDS), 36.7 million people were living with HIV globally by end of the year 2016, yet 30% did not know their status [1, 2]. Majority of the people living with HIV/AIDS come from low and middle income countries with 25.5 million living in Sub-Saharan Africa. Globally, the number of new infections has declined from 2.1 m in 2015 to 1.8 m in 2016 [1, 2]. The number of people receiving HIV treatment has also drastically increased over the recent years, particularly in resource-limited countries. By the end of 2016, more than half (53%) of the people living with HIV/AIDS had access to treatment [1, 2]. Although countries have made significant progress against HIV/AIDS, the efforts are not fast enough to achieve the global 90:90:90targets for HIV elimination [3].

According to the viral load dash board-Uganda as of November 2018, 78% of all PLHIV know their status, 86% of all PLHIV have been enrolled into care, and 77% of all persons on ART are virally suppressed but this varies by region and age category. However, the current dash board does not take care of the different categories of key populations (KP) including female sex workers (FSW) but they should be far below the current global target of HIV elimination because of poor health seeking behavior due to their being highly mobile and unconducive legal environment in Uganda.

By end of 2016, 1.4 million people were living with HIV in Uganda. The adult HIV prevalence was 6.2% (those aged 15 to 64 years) [4]. Women were more disproportionately affected with 7.6% of adult women in Uganda living with HIV/AIDS compared to the men at 4.7% [5]. Among adults living with HIV who reported knowledge of their HIV status, 58.5% reported current use of ART: 61.9% of HIV positive women and 52.4% of HIV positive men [5]. The Uganda AIDS Commission (UAC, 2014) estimated over 54,549 female sex workers in Uganda and the National HIV Investment Case (2015–2025) indicates an HIV prevalence of 35% while UNAIDS (2014) estimates the prevalence at 34.2%. The high HIV risk among sex workers arises from high rates of unprotected sex, alcohol, drug use, and non-use of condoms. This is coupled with the discriminatory laws to sex work and the limited capacity to bargain for the right to social protections among others [6].

Key populations continue to lag behind the general population in achieving 90–90-90 HIV outcomes. Current estimates suggest that 47% of all continuing HIV transmissions globally are driven by KPs and their sexual partners (UNAIDS, 2018). Among the FSWs, the rate of new HIV infections was 13 times greater than in the general population (UNAIDS special analysis, 2018). Similarly, the risk of acquiring HIV for people who inject drugs (PWID) was 22 times higher than for people who do not inject drugs, and 13 times higher for transgender (TG) women than adults aged 15–49 years (UNAIDS. Miles to Go Global AIDS Update, 2018). In Uganda’s HIV epidemic context, KPs at high risk of HIV constitute the following population categories: Men who have Sex with Men (MSM), FSW, PWID, People in closed setting (Inmates and prisoners), Unifromed forces, and TG (UAC, 2015). A FSW is any adult who engages in the act of exchanging money for sexual services with a client. This involves negotiation and exchange of money for a sexual service and it is a regular income-generating practice that can take place anywhere. A FSW can work independently as individuals (on the streets) or for a company. They may also be managed by a pimp, or work as part of a brothel and bars among others.

In Uganda, FSWs are markedly underserved and marginalized by HIV prevention programmes, they face sexual violence associated with Sexual Gender Based Violence (SGBV) from clients due to failure to pay the agreed amount of money or having sex without a condom and yet it was not agreed upon during the initial negotiations. They also have concurrent multiple sexual partners, they practice risky sex with limited access to condoms and treatment of sexually transmitted infections (STI). Drug-use is also common among the FSWs which could result in impaired judgment of risky sexual behaviours. Addressing such challenges is critical to the success of HIV care programs.

In Uganda, HIV testing is conducted in health facilities, community settings, and homes. Over the recent past, emphasis has been placed on strategies such as workplaces testing, outreach to most at risk groups, and mobile or mass testing, especially during testing campaigns [6]. HIV testing for FSWs is currently being done at the static health facilities, in the community at the drop in centers or during the outreaches and self-testing in some parts of the country is being offered for sex workers through their peers or health workers. The number of people accessing HCT has increased over time from 5.1 million in 2012 to 10.3 million in 2015 [6].

In Uganda, sex work is illegal. Therefore, because of its criminalization and associated social stigma, FSWs do not access HIV health care services and if they do, they conceal their occupation from health care workers. It was estimated that FSWs and their clients accounted for 18% of the new HIV infections in 2015/2016 [6]. Given the impact of the FSWs’ high HIV prevalence on the overall HIV prevention and care efforts, special interventions have been designed to address the challenge among the various categories of key populations including FSWs. Specific community-based HIV testing service delivery models such as static facilities, outreaches through moonlight services, outreaches to safe spaces, bars, clubs, lodges, brothels, and Camps (using a mobile van), and peer to peer mechanism have been designed and implemented. Despite the efforts by Ministry of Health Uganda (MoH) and patterns to reach FSWs, very few FSWs have been reached with the HIV prevention interventions and the HIV prevalence has remained high among this category of key population. We assessed the preference and uptake of the current community-based HIV testing services delivery models that are used to reach FSW along Malaba-Kampala high way and identified challenges faced during the implementation of the models., a Malaba-Kampala high way, is a major busy high way from Mombasa-Kenya that links Uganda to other countries such as Rwanda, Tanzania, and Burundi with many hotspots and identified challenges associated with the models. Based on the assessment, we identified answers to the following research questions: What is the preference and uptake of different current community-based HIV testing services delivery models among FSWs along Malaba- Kampala highway? and what are the challenges in the different community-based HIV testing service delivery models used to reach FSWs along Malaba- Kampala highway?

## Methods

### Study setting

We conducted the study at hot spot areas along the Malaba-Kampala highway in six districts namely, Busia, Tororo, Bugiri, Iganga, Jinja, and Kampla. We defined hot spots as geographical locations where high risk activities such as pick-up points or places where the actual execution of sex work takes place or are concentrated. The hot spot areas included: Malaba-Tororo highway, Malaba-Busia highway, Iganga, Bugiri, Jinja, and Kampala. We selected Malaba-Kampala highway based on the fact that it is one of the major highways in Uganda where a lot of sex work occurs.

### Study design

We conducted a cross-sectional study using both quantitative and qualitative data collection methods: We assessed the preference and uptake of the different community-based HIV testing service delivery models based on two indicators, that’s, the proportion of FSWs who had an HIV test in the last 12 months (June 2015–July 2016) and the proportion of FSWs who were positive and linked into care (Table 1).

**Table 1:** Indicators used to assess the preference and uptake of different community-based HIV testing service delivery models

HIV testing by female sex workers in the last 12 months	
Definition of the indicator	The proportion of female sex workers who have had an HIV test in the last 12 months and know the results through the different community model
Target	All sampled female sex workers along Malaba-Kampala highway
Numerator	The number of female sex workers who have had an HIV test in the last 12 months and know their status.
Denominators	All female sex workers interviewed
HIV Positive female sex workers immediately linked to care through the different model	
Definition of the indicator	The proportion of HIV positive female sex workers linked to care in the last 12 months through the different models. Immediate linkage means a newly identified HIV+ female sex worker who has been referred and enrolled into care. It is recommended that an HIV + individual is enrolled within 7 days at the same health facility of identification or within 30 days if referred to another health facility
Target	All positive HIV female sex workers who tested in the different models
Numerator	The number of positive HIV female sex workers linked to care by the different models
Denominator	All HIV positive female sex workers who tested in the different models

### Description of the community-based HIV testing sservice delivery models

Ministry of Health Uganda provides the policy guidance and standards for health services and facilities. Ministry of Health Uganda acknowledges that HIV programmes for Most At Risk Populations (MARPs) along the transport corridors are critical in prevention and control of HIV. The community-based HIV testing models that target FSWs were established in 2009 and these include: Static facility services (clinics in hot spot areas), outreaches, and peer to peer mechanism.

#### Static facility based services

These are located along hotspot areas where FSWs can easily go and access HIV testing services without incurring high travel costs. They are stocked with free commodities like condoms, water based lubricants, HIV testing kits, STI drugs, and supplies for HIV/AIDS care. These facilities are open daily and have at least three service providers. These health workers are informed and are in contact with referral health facilities for the services they cannot offer. Female Sex workers consult service providers without any appointment through centre visits or phone calls.

#### Outreach posts and sites

Services are provided in a variety of settings in the community, either as outreach to community sites or through mobile vans or tents. Each of the health facility serving a given hot spot area makes an annual worker plan and health workers move out to these hot spots at least once a week. The team has got at least four health workers that include a clinician, nurse, lab personnel, counselor, and the mobilization is done by the peers. These services are temporary, but regular in community sites where FSWs operate and reside such as entertainment centers like bars, clubs, and lodges. Extra space is created by provision of tents or mobile vans stationed in high risk places. Outreach posts and sites on average operate 4 to 8 days in a month.

#### Peer to peer mechanism

Here, two FSWs are selected by their colleagues, trained and equipped with knowledge on HIV and skills to reach peers by Most-At-Risk-Population Initiative (MARPI) or any comprehensive HIV partner who is in charge of the region such as The Aids Support Organization (TASO), Regional Health Integration to Enhance Services in East Central Uganda (RHITE-EC) with the guidance of the district and health facility where the peers are attached. The trained FSWs then share information provided to them by the health workers, encourage their colleagues to seek HCT services, distribute condoms, and link them to care. These Peers are paid for the mobilization done by the responsible pattern like MARPI through the heath facility staff or the focal person for KPs to which the peers are attached.

HIV Counselling and Testing services are being provided through drop in centre (safe space either in the community or attached to the facility which key population like Female Sex Workers use as entry point to access services or a congregation point to discuss issues pertaining to their health and welfare and other social issues)) which are located in the hot spot, outreaches are being carried to hot spot areas which include bars, brothels, and the peers do provide HCT testing using the self testing kits. HIV Counselling and Testing services are being provided by the public health facilities that have the drop in centres. The government of Uganda provides the testing kits and NGO & CSO such as MARPI, TASO, Infectious Diseases Institute (IDI) with funding from President’s Emergency Plan For AIDS Relief (PEPFAR) or Global Fund pays allowance for the health workers, Peers, and facilitation for transport and space during outreaches.

The community-based HIV/AIDS service delivery models along Malaba-Kampala highway are being provided by local governments in partnership with non-government organizations (NGO) and community-based organizations (CBO) such as MARPI, Family Health International (FHI), Aids Information Center (AIC), Uganda Reproductive Health Bureau (URHB), Malaba Kyosimba Onaamya Community Development Association (MAKOCDA), Reach Out Mbuya, IDI. These are funded by PEPFAR or Global fund and are coordinated by the MARPs Network which was formed in 2007 by MoH in partnership with the Uganda Health Marketing group (UHMG).

#### Study population

We conducted the study among FSWs aged ≥18 years who operated along the Malaba-Kampala highway. We defined SWs as any female aged 18 years and above, who undertakes sexual activity after consenting with a man for money or other items/ benefits as an occupation or as a primary source of livelihood irrespective of site of operation within the last 6 months i.e. street, bars, home, hotel, and other locations and they should have operated along Malaba-Kampala high way for at least 1 year. We interviewed health service providers from six health facilities as key informants which included clinicians, counselors, nurses, and pharmacists at points of care, district political leaders (Resident District Commissioners (RDC) and Secretaries for Health), technical leaders such as District Health Officers or HIV focal persons and focal persons for key populations of Implementing Partners (IP) in all the six districts, and Ministry of Health. At the MoH Uganda level, we engaged the AIDS Control Program (ACP), Uganda AIDS Commission (UAC), and other partners such as, AIDS Information Center (AIC), Uganda Reproductive Health Bureau (URHB). The IPs engaged included MAKOCDA, Reach Out Mbuya, IDI, Mildmay Uganda, TASO Uganda, and the MARPs Network.

### Sample size and sampling considerations

Hot spots and Sex workers: We used cluster sampling for hot spot selection. We purposively selected the “hot spots” along the Malaba-Kampala highway. In each hot spot, all the popular bars, streets, and brothel where FSWs operated were taken as a cluster in that district. The actual population of FSWs in the different clusters (hot spots) was not known, so we assumed an equal population in all the selected clusters. The sample size for this cross-sectional study was determined using the Kish leslie formula (Kish leslie, 1965) for single proportion. We recruited a total of 72 FSWs in each cluster (420 FSWs in total) determined based on the calculation. Because FSWs are a hard-to-reach category, we used snowballing approach to reach the 72 needed for each cluster (Ritchie, 2003). We only included FSWs who were residents within the programs (community-based HIV testing service delivery models) defined catchment areas. These FSWs had to have spent ≥ 1 year in the defined catchment area for community-based HIV testing service delivery model prior to the date of interview. This was a consideration for both qualitative and quantitative data collection methods, the participates were asked if they had received HIV services in the past 12 month. Female sex workers who were found to be too sick to respond as judged by the research assistants (RA) and eligible participants who did not consent were excluded from the study.

Service providers, Ministry of Health Uganda, and Implementing Partners: We purposively selected service providers in health facilities that were providing services to FSWs and these included clinicians, counselors, nurses, and pharmacists who worked directly with SWs at selected health facilities within the six districts along the Malaba-Kampala highway. In total, we recruited 12 service providers from six health facilities that were providing services for FSWs in the selected districts. We also purposively selected respondents from MoH Uganda (ACP = 2), IPs including technical officers (MARPs, AIC, Reach Out Mbuya, IDI, Mildmay, and TASO **=** 12), Politicians (RDCs, and Secretaries for Health = 6), and patrons = 6. We conducted a total of 6 FGDs with SWs (1 at every hot spot). The qualitative questions asked during the assessment focused on factors affecting implementation and suggestions on how to effectively implement HIV services models that are used to reach FSWs. We also explored the experiences, challenges, acceptability of community-based HIV testing service delivery models used to reach FSW.

### Data collection

We recruited Research Assistants (RAs) who then recruited FSWs in the company of local contact persons such as peer FSWs, bar owners or brothel managers. We defined local contact persons as a member of the community who had regular interaction with FSWs, peer FSWs or a patron of a bar and other entertainment venues that FSWs usually frequented. The RAs were fluent in both English and the study area local languages and also had prior experience of working with FSWs. We used a semi-structured questionnaire and data abstraction tools (Supplementary material) to obtain information on the proportion of FSWs who had an HIV test in the last 12 months and those who were positive and linked into care under the different models mainly by self report or reviewing their medical record if available. We recruited both male and female RAs to cater for female SWs who were more comfortable with the opposite sex interviewing them. For the busy FSWs, we requested for appointments to match their convenient time.

In-addition, we assessed the respondents’ views on the challenges and factors which are crucial for the success of community-based HIV testing service delivery models using key informant interviews and focus group discussions. We translated the data collection tools into relevant local languages and back translated by other people without prior knowledge of the instrument in order to maintain the original meaning of the questions.

### Data management and analysis

We entered and analyzed quantitative data in Epi-info 7. We used descriptive statistics to summarize participants’ demographic characteristics. Categorical variables were described using frequencies and percentages, while continuous variables were summarized using means (±SD) or medians (IQR).

For qualitative data, raw data from FGDs and KIIs– were recorded, translated, transcribed and typed in detail. In addition, field diaries from the data collection process, with records of any events deemed important for the interpretation of the results were also typed out and integrated for the data interpretation and analysis stage. After the data transcripts had been typed, we read through repeatedly to identify the codes and the emerging themes.

We analyzed qualitative data using the Thematic and Template Analysis (TA) approach (King, 2012; Braun & Clarke, 2006). For data management, we used the NVivo 10 software. Guided by the study objectives, the typed data transcripts were entered into the NVivo 10 software after which they were assigned specific codes that related to the theme or pattern they fell under (the template approach). Following this, we then reviewed and related the multiple emerging themes related to the study objectives and research questions for analysis to begin. In addition to use of the software, analysis for qualitative data was further guided by data from the field diaries which helped contextualize the data from verbal/audio sources. The process of coding took place in six phases to create established and meaningful patterns. These phases were: familiarization with data, generating initial codes, searching for themes among codes, reviewing themes, defining and naming themes, and finally writing the report.

## Results

### Socio-demographic characteristics of respondents

The majority 61% (279/456) of the FSWs belonged to age category 25–34. Female sex work was the primary occupation (source of lively hood) for almost all 99% (453/456) of the FSWs. Half, 51% (234/456) of the FSWs reported to have been divorced or separated or widowed from their partners. Forty eight percent (219/456) of the FSWs had attained primary education while only, 34.2% (156/456) had secondary education. The majority, 72% (327/456) of the FSWs were permanent residents of the place of interview (Stayed in the study area for at least 1 year) (Table 2).

**Table 2:** Socio-demographic characteristics of the respondents (*N* = 456)

Characteristic	Frequency	Percentage
Age		
18–24	102	22.4
25–34	279	61.2
35+	75	16.4
Primary occupation		
Sex workers	453	99
Hair dresser	3	1.0
Religion		
Christians^a^	294	64.5
Moslems	159	34.9
Others	3	0.7
Education		
None	75	16.4
Primary	219	48.0
Secondary	156	34.2
Post secondary	6	1.3
Marital status		
Single	120	26.3
Married/ (cohabiting)	102	22.4
Divorced/separated/widowed	234	51.3
Residence in relationship to the area of operation were a FSW was found and interviewed		
Permanent	327	71.7
Temporary	48	10.5
Mobile	81	17.8
Amount of money earned per day		
10,000/= to 20,000/=	90	19.7
21,000/= to 30,000/=	234	51.3
31,000/= to 40,000/=	48	10.5
41,000/= to 50,000/=	57	12.5
51,000/= to 60,000/=	15	3.3
61,000/= and above	12	2.6

^a^(Protestants, Catholics & Seventh Day Adventists)

### Preference and uptake of community-based HIV testing service delivery models by female sex workers, Malaba-Kampala higway, Uganda, June 2015–July 2016

Overall, the majority, 85.5% (390/456) of the FSWs had taken an HIV test in the last 12 months. Of the 390 FSWs, the majority, 72% (279/390) had used static facilities, 25% (98/390) had used outreaches, and 3.3% (13/390) used peer to peer mechanisms to have an HIV test. Overall, 35% (159/390) of the FSWs who had taken an HIV test were HIV positive. Of the 159 FSWs who were HIV positive, the majority, 83.0% (132/159) were linked into care. Of the 132 SWs linked into care, the majority, 81.8% (108/159) were linked into care immediately that is to say within a period of less than a month. All most all, 91% (120/132) of the FSWs reported to have been linked into care by static facilities. Of the 108 FSWs linked into care immediately, more than half, 69.4% (75/108) were linked through static facilities. Of the 24 FSWs linked to care after delay, the majority, 75% (18/24) were linked through the static facilities. Of the FSWs linked into care, the majority, 88.6% (117/132) were on ART and the rest had access to ART related drugs or were registered in ART clinics (Table 3).

**Table 3:** Preference and uptake of the different HIV testing services delivery models by female sex workers along Malaba-Kampala highway, Uganda

Variable	Frequency	Percentage
Number of FSWs that received HCT in the last 12 months	*N* = 456	
Overall	390	85.5
Source of HCT services in the last 12 months	*N* = 390	
Static facilities	279	71.7
Outreaches	98	25
Peer to peer mechanism	13	3.3
HIV status	*N* = 456	
Positive	159	35
If positive are linked into care	*N* = 159	
Overall	132	83
If linked into care what was the estimated period	*N* = 132	
Immediately (within one month)	108	82
Static	75	69.4
Outreach	29	26.7
Peer to peer mechanism	4	3.7
Delayed	24	18
Static	18	75
Outreach	4	16.7
Peer to peer mechanism	2	8.3
Mechanism used for linkage into care	*N* = 132	
Static	120	90.1
Outreach	9	6.8
Peer to peer mechanism	3	2.3
Linked into care and on ART	*N* = 132	
Yes	117	88.6
Static	109	93.2
Outreach	6	5.1
Peer to peer mechanism	2	1.7
No	15	11.4
Static	11	8.3
Outreach	3	2.3
Peer to peer mechanism	1	0.8
Status for those linked into care but not on ART		
Registered in a clinic	3	20
Registered and still active	6	40
Access to ARV related drugs	6	40

### Challenges in the current community-based HIV testing service delivery models used to reach female sex workers along Malaba- Kampala highway, Uganda, June 2015–July 2016

#### Challenges faced by service providers

Service providers were faced with stock out of test kits to meet the demand of HCT services. This happened when there was good mobilization and many people turn up for testing especially during outreaches. This challenge was especially observed where mobilization was done by an NGO and testing kits were provided by government health facilities (Table 4).

*“This happens when you mobilize people during outreaches and many people turn up especially if there is a promotional function, we test and run out of testing kits and we only refer them to where they can have an HCT service from during the day but you know, if a person has taken a decision to take an HCT and she/he does not have it then, it will take much more time to get that courage of testing a gain being that this is a mobile community who are so active in the night and during the day when the clinics where we refer them are open they are also sleeping” (KI Busia).*

At the facility level, there were periods reported when they were no test kits; the few available were reserved for pregnant women. So sometimes, FSWs and other people were told to come back later, those who could afford to pay would go to private clinics (Table 4).

*“At times people come to our facility and they wish to test and we tell them to come back when we have testing kits or refer them to private clinics who take advantage and charge them 10,000/= ($2.7), this is too much yet people do not have food. If government can increase on the supply in government health facilities or even partner with other organizations and they are supplied cheaply just like the malaria testing kits” (Health worker in one of the clinic in hot spot areas.)*

Our respondents reported a number of challenges associated with outreaches. Some of the business owners did not want health workers to carry out HIV testing at their premises as this would disrupt their business. Unwillingness to allow health workers conduct HIV testing at their bars demonstrates the perception about HCT outreaches that are carried out during the night at bars (Table 4).

*“If somebody has come to have fun and he or she is persuaded to take an HIV test and the results are not good they will just go back home and if that person is with a sex worker and the sex worker is positive he will abandon her and instead of taking like six bottles of beer they will end up taking three because he is now alone”*

*“Then there are people who know or doubt their status, they will not come to our bars if they hear that there is HIV testing going on. So this is a loss to the business” (Bar owner -Jinja).*

Some people do not trust results given during outreaches especially if blood is picked from a finger. People still think that a lot of blood should be used for HIV testing and the testing should be done in a laboratory with a microscope. The quote below demonstrates the misconception that the SWs had about HCT services provided during outreaches and worse if blood is picked from a finger:

*“Some clients use phones to test for blood pressure and HIV, so when you give them results which are different from what they got using their phones and you picked blood from a finger, it will be hard for those people to trust the results given” (KI Tororo)*

**Table 4:** Challenges faced by service providers and female sex workers within different HIV testing service delivery models along Malaba-Kampala highway, Uganda

Category of individuals	Challenges faced
Service providers/health workers	Service providers were faced with stock out of testing kits to meet the demand of HCT services. This was reported to happen when there was good mobilization and many people turn up for testing especially during outreaches. This challenge was especially observed where mobilization was done by an NGO and testing kits were provided by government health facilities.
	At the facility level, some periods were reported when they were no testing kits; the few available were reserved for pregnant women. So sometimes female sex workers and other people were told to come back later, those who could afford to pay would go to private clinic
	A number of problems were found to be associated with outreaches. Some of the business owners did not what health workers to carry out HIV testing at their premises as this would disrupt their business
	Some female sex workers do not trust results given during outreaches especially if blood is picked from a finger. People still think that a lot of blood should be used for HIV testing and the testing should be done in laboratory with a microscope
	Most outreaches are irregular and mainly depend on the availability of funds and testing kits. This always make linkage into care very impossible due to the fact that female sex workers are mobile and move depending on availability of clients and sometime they move with their clients.
Female sex workers	Female sex workers also faced challenges during the provision of HIV service to them. Most of them report long waiting hours especially in static clinics where services were provided to them together with the general population. This made most of them to miss HCT and other related services.
	Female sex workers also reported that most of the places do not have specific clinic for female sex workers making services provided to them being not user friendly. In most of the “hot spot” areas visited there was only one specific clinic serving female sex workers and the few available clinic had no funder which had lowed service provision to this category of people
	Stigma and discrimination is still high among female sex workers especially from the health workers who have not been trained in provision of friendly services for KPs, this was one of the reason that was reported by female sex workers not taking HCT services
	Female sex workers also complained about low or lack facilitation for peer leaders. These do voluntary worker and most of the time they are not compensated for their work yet they lose time and customers when doing this.
	The quality of services was reported to be poor due to the fact that there was no holistic service provided to female sex workers during outreaches. They suggested for inclusion of others services during outreaches such as tests for Hepatitis B, syphilis etc. This would improve on the quality of services provided to them and would encourage other colleagues to come because of the variety of the service that they expect to receive as shown by the quote.

Another example of a misconception arose when some of the SWs got results that are different from what they know or what they think (Table 4).

*“They should improve because when they come to test you they tell you that you are negative. So when you go to another government facility they tell you that you are positive. For me I have started taking drugs in TASO but when they test me they give me negative results yet the virus is just dormant because I have taken drugs for a long time” (*FGD with FSWs in Malaba-Tororo district)

Most outreaches are irregular and mainly depend on the availability of funds and test kits. This always makes linkage into care very difficult due to the fact that SWs are mobile and move depending on availability of clients and sometimes they move with their clients as demonstrated by a KI from Iganga (Table 4).

*“When you take a month to go back to the community, linkage becomes a challenge because this is a mobile community and naturally if people are positive they have many questions which need a lot of time to be answered and there is need for the clients to be in touch with the health workers during that period so this is not possible especially where you do not have a specific period when you will go back to the community. When you go back you will meet different people who will come for testing” (*KI Iganga)

#### Challenges faced by female sex workers

Female sex workers also faced challenges while accessing and receiving HIV/AIDs care services. Most of them report long waiting hours especially in static clinics where services were provided to them together with the general population. This made most of them to miss HCT and other related services as demonstrated by the FGDs (Table 4).

*“So many people do not want to wait for so long in a line. There is no specific person specially in HCIIs who is trained to offer SW friendly services so we have to wait in the line until your chance comes up and sometimes if you get a customer you leave and go and attend to the customer and you may never go back and even if you went back you may be told to come back the following day because the health worker is tired” (*FGD with FSWs in Bugiri).

Female sex workers also reported that most of the places do not have specific clinics for them (FSWs) making services provided being not user friendly. In most of the “hot spot” areas visited there was no specific clinic serving FSWs and the few available clinics had no funder which had limited service provision to this category of people as demonstrated by the FGD with FSWs in Jinja (Table 4).

*“When you go to these facilities to seek services and you tell them that you are a FSW they do not handle you well after learning that you are a FSW, health workers can start discussing about you and looking at you and yet we are also human beings it’s just that we want to survive otherwise we also do not like our work” (*FGD with FSWs Jinja)

Stigma and discrimination is still high among FSWs leading to failure to access and utilize HCT services. As reported by most FSWs (Table 4).

*“When you test with your colleagues and they get to know your results they will tell other people and you may end up losing customers. That is why I do not test from here, I wait when I have gone back home that is when I test from private clinics” (*FGD with FSWs in Iganga)

*“We do not want to be seen getting treatment here, we prefer to get treatment from far where people do not know us, otherwise you may lose customers when they know that you are positive” (*FGD with FSWs in Busia)

The FSWs also complained about low or lack of facilitation for peer educators. These do voluntary work and most of the time they are not compensated for their work yet they lose time and customers when doing this (Table 4).

*“We have no facilitation while doing this peer to peer work and yet even our fellow FSWs think we get a lot of money and they want us to give them some money before we talk to them. You people are paid and you earn a lot of money from us, they retort”* (FGD with FSWs in Tororo).

The quality of services was reported to be poor due to the fact that there was no holistic service provided to FSWs during outreaches. They suggested for inclusion of other services during outreaches such as Hepatitis B and syphilis testing among others. This would improve on the quality of services provided to them and would encourage other colleagues to come because of the variety of services that they expect to receive (Table 4).

*“If they organize these outreaches we also need to get other services such as tests for syphilis, Hepatitis B, etc. since these diseases are quite common among us FSWs”* (FGD with FSWs in Bugiri).

## Discussion

In this study conducted to assess the preference and uptake of community-based HIV testing service delivery models to FSWs, most of the FSWs had HCT services and were linked to care through static facilities compared to outreaches, and peer to peer mechanisms. Among the three community-based HIV testing service delivery models, FSWs preferred static facilities. Static facilities were preferred for reasons such as ensuring patient confidentiality, and services always being available whenever FSWs desired to have them. Despite the better performance of static facilities, they were also faced with challenges such as long waiting times to obtain services which discouraged some FSWs from accessing HIV testing services.

The limited performance of outreaches and peer to peer mechanisms could be attributed to business owners not willing to have such services conducted in their premises as it would disrupt business. Outreach services were also characterized by lack of trust in the results generated, irregular service provisions, and failure to offer all demanded services such as hepatitis B and syphilis testing to the FSWs.

However, all the models were faced with stock out of HIV test kits, failure to offer FSW friendly services, and stigma and discrimination among the FSWs. In order to reach the population-level impact of HIV prevention, HIV/AIDS programs among FSWs should also offer comprehensive services including how to overcome stigma and discrimination, FSW friendly, and sexually transmitted infection (STI) services. Health service providers should therefore strive to offer comprehensive and integrated health programs for FSWs [7]. Additionally, there is need to implement the MoH Uganda STI/HIV prevention action plan that recommends screening and treating of all STIs among the MARPs including FSWs [8].

According to a review conducted by Wilson, HIV/AIDS care programs for FSWs in low and middle income countries lack consistency and quality [7]. Lack of consistency of outreach programs according to health care providers and documented literature may be due to the fact that most of such programs in low and middle income countries are donor dependent and are therefore prone to such challenges [7, 9, 10]. Irregular provision of services makes it hard for health workers to be in touch with the FSWs hence making HIV testing and linkage to care a problem. This calls for local political will and increased domestic funding to ensure increased uptake of the FSW HIV testing programs.

The proportion of FSWs who had taken an HIV test is still below the 90–90-90 UNAIDS targets for HIV elimination where we want to have 90% of the people knowing their HIV status [11]. The prevalence of HIV among the SWs reported by our study is consistent with the national prevalence at 35% [12]. This prevalence is still high compared to the general populations’ at 6.2% [11]. Linkage to care is important, of those who were HIV positive, majority were linked to care and more than 90% were on ART. The current national antiretroviral treatment guidelines require that every person including FSW found to be HIV positive should start ART regardless of their CD4 [13].

Peer educators also reported poor facilitation or no facilitation. For interventions that target FSWs to be successful, there is need to compensate peer educators for the time spent offering services to colleagues in the same business.

### Limitations

Our findings should be interpreted based on the biases that are inherent in sampling hard to reach populations and respondent driven sampling. We paid $4 to each of the FSWs as an incentive to participate in the study and also compensate for losses while interacting with the RAs. Given the generally poor economic status of most Ugandans, and the fact that most of the FSWs reported earning $3 or less per transaction act, it is possible that some of the respondents were not FSWs but other categories of persons attracted by the incentive attached to the study. However, chances of recruiting none FSWs were reduced by using RAs who had prior experience in working with the FSW population in Uganda. We also tested the incentive amount while piloting the study tool. Additionally, our study only focused on the number of FSWs who benefited from the different models. We did not do a cost analysis of how much it would cost to reach each female sex worker using the different models.

## Conclusions and recommendations

All the three service delivery models were being utilized by the FSWs. However, most of the FSWs had HCT services and were linked to care through static facilities compared to the outreaches, and peer to peer mechanism. Community-based HIV testing service delivery models are challenged with inconsistent flow of HIV diagnostics and supplies and unfriendly services. We recommended strengthening of the all the HIV testing models by ensuring constant supply of test kits, other necessary supplies, opening up facilities that offer FSW friendly services, and provision of a comprehensive HIV care package to the FSWs.

## Data Availability

The datasets used and analyzed during this study belong to the Uganda Public Health Fellowship Program. However, they are available from the corresponding author on reasonable request and upon permission from the Uganda Public Health Fellowship Program.

## Abbreviations

ACP: AIDS Control Program
AIC: AIDS Information Center
ART: Antiretroviral therapy
ARV: Antiretroviral drugs
CD4: Cluster of differentiation 4
CDC: US Centers for Disease Control and Prevention
FGD: Focus Group Discussion
FHI: Family Health International
FSW: Female Sex Worker
HCT: HIV counseling and testing
HIV: Human immunodeficiency virus
HIV/AIDS: Human immunodeficiency virus/Acquired immunodeficiency syndrome
IDI: Infectious Diseases Institute
KI: Key Informant
KII: Key Informant Interview
MAKOCDA: Malaba Kyosimba Onaamya Community Development Association
MARPS: Most At Risk Populations
NGO: Non-government organizations
RA: Research Assistant
RDC: Resident District Commissioner
TASO: The Aids Support Organization
UAC: Uganda AIDS Commission
UHMG: Uganda Health Marketing Group
URHB: Uganda Reproductive Health Bureau
WHO: World Health Organization
